# Large-scale Analysis of Counseling Conversations: An Application of Natural Language Processing to Mental Health

**Published:** 2016

**Authors:** Tim Althoff, Kevin Clark, Jure Leskovec

**Affiliations:** Stanford University

## Abstract

Mental illness is one of the most pressing public health issues of our time. While counseling and psychotherapy can be effective treatments, our knowledge about how to conduct successful counseling conversations has been limited due to lack of large-scale data with labeled outcomes of the conversations. In this paper, we present a large-scale, quantitative study on the discourse of text-message-based counseling conversations. We develop a set of novel computational discourse analysis methods to measure how various linguistic aspects of conversations are correlated with conversation outcomes. Applying techniques such as sequence-based conversation models, language model comparisons, message clustering, and psycholinguistics-inspired word frequency analyses, we discover actionable conversation strategies that are associated with better conversation outcomes.

## 1 Introduction

Mental illness is a major global health issue. In the U.S. alone, 43.6 million adults (18.1%) experience mental illness in a given year (National Institute of Mental Health, 2015). In addition to the person directly experiencing a mental illness, family, friends, and communities are also affected (Insel, 2008).

In many cases, mental health conditions can be treated effectively through psychotherapy and counseling (World Health Organization, 2015). However, it is far from obvious how to best conduct counseling conversations. Such conversations are free-form without strict rules, and involve many choices that could make a difference in someone’s life. Thus far, quantitative evidence for effective conversation strategies has been scarce, since most studies on counseling have been limited to very small sample sizes and qualitative observations (*e.g.*, Labov and Fanshel, (1977); Haberstroh et al., (2007)). However, recent advances in technology-mediated counseling conducted online or through texting (Haberstroh et al., 2007) have allowed counseling services to scale with increasing demands and to collect large-scale data on counseling conversations and their outcomes.

Here we present the largest study on counseling conversation strategies published to date. We use data from an SMS texting-based counseling service where people in crisis (depression, self-harm, suicidal thoughts, anxiety, *etc.*), engage in therapeutic conversations with counselors. The data contains millions of messages from eighty thousand counseling conversations conducted by hundreds of counselors over the course of one year. We develop a set of computational methods suited for large-scale discourse analysis to study how various linguistic aspects of conversations are correlated with conversation outcomes (collected via a follow-up survey).

We focus our analyses on counselors instead of individual conversations because we are interested in general conversation strategies rather than properties of specific issues. We find that there are significant, quantifiable differences between more successful and less successful counselors in how they conduct conversations.

Our findings suggest actionable strategies that are associated with successful counseling:

1. **Adaptability (Section 5):** Measuring the distance between vector representations of the language used in conversations going well and going badly, we find that successful counselors are more sensitive to the current trajectory of the conversation and react accordingly.
2. **Dealing with Ambiguity (Section 6):** We develop a clustering-based method to measure differences in how counselors respond to very similar ambiguous situations. We learn that successful counselors clarify situations by writing more, reflect back to check understanding, and make their conversation partner feel more comfortable through affirmation.
3. **Creativity (Section 6.3):** We quantify the diversity in counselor language by measuring cluster density in the space of counselor responses and find that successful counselors respond in a more creative way, not copying the person in distress exactly and not using too generic or “templated” responses.
4. **Making Progress (Section 7):** We develop a novel sequence-based unsupervised conversation model able to discover ordered conversation stages common to all conversations. Analyzing the progression of stages, we determine that successful counselors are quicker to get to know the core issue and faster to move on to collaboratively solving the problem.
5. **Change in Perspective (Section 8):** We develop novel measures of perspective change using psycholinguistics-inspired word frequency analysis. We find that people in distress are more likely to be more positive, think about the future, and consider others, when the counselors bring up these concepts. We further show that this perspective change is associated with better conversation outcomes consistent with psychological theories of depression.

Further, we demonstrate that counseling success on the level of individual conversations is predictable using features based on our discovered conversation strategies (Section 9). Such predictive tools could be used to help counselors better progress through the conversation and could result in better counseling practices. The dataset used in this work has been released publicly and more information on dataset access can be found at http://snap.Stanford.edu/counseling.

Although we focus on crisis counseling in this work, our proposed methods more generally apply to other conversational settings and can be used to study how language in conversations relates to conversation outcomes.

## 2 Related Work

Our work relates to two lines of research:

### Therapeutic Discourse Analysis & Psycholinguistics

The field of conversation analysis was born in the 1960s out of a suicide prevention center (Sacks and Jefferson, 1995; Van Dijk, 1997). Since then conversation analysis has been applied to various clinical settings including psychotherapy (Labov and Fanshel, 1977). Work in psycholinguistics has demonstrated that the words people use can reveal important aspects of their social and psychological worlds (Pennebaker et al., 2003). Previous work also found that there are linguistic cues associated with depression (Ramirez-Esparza et al., 2008; Campbell and Pennebaker, 2003) as well as with suicude (Pestian et al., 2012). These findings are consistent with Beck’s cognitive model of depression (1967; cognitive symptoms of depression precede the affective and mood symptoms) and with Pyszczynski and Greenberg’s self-focus model of depression (1987; depressed persons engage in higher levels of self-focus than non-depressed persons).

In this work, we propose an operationalized psycholinguistic model of perspective change and further provide empirical evidence for these theoretical models of depression.

### Large-scale Computational Linguistics Applied to Conversations

Large-scale studies have revealed subtle dynamics in conversations such as coordination or style matching effects (Niederhoffer and Pennebaker, 2002; Danescu-Niculescu-Mizil, 2012) as well as expressions of social power and status (Bramsen et al., 2011; Danescu-Niculescu-Mizil et al., 2012). Other studies have connected writing to measures of success in the context of requests (Althoff et al., 2014), user retention (Althoff and Leskovec, 2015), novels (Ashok et al., 2013), and scientific abstracts (Guerini et al., 2012). Prior work has modeled dialogue acts in conversational speech based on linguistic cues and discourse coherence (Stolcke et al., 2000). Unsupervised machine learning models have also been used to model conversations and segment them into speech acts, topical clusters, or stages. Most approaches employ Hidden Markov Model-like models (Barzilay and Lee, 2004; Ritter et al., 2010; Paul, 2012; Yang et al., 2014) which are also used in this work to model progression through conversation stages.

Very recently, technology-mediated counseling has allowed the collection of large datasets on counseling. Howes et al. (2014) find that symptom severity can be predicted from transcript data with comparable accuracy to face-to-face data but suggest that insights into style and dialogue structure are needed to predict measures of patient progress. Counseling datasets have also been used to predict the conversation outcome (Huang, 2015) but without modeling the within-conversation dynamics that are studied in this work. Other work has explored how novel interfaces based on topic models can support counselors during conversations (Dinakar et al., 2014a; 2014b; 2015; Chen, 2014).

Our work joins these two lines of research by developing computational discourse analysis methods applicable to large datasets that are grounded in therapeutic discourse analysis and psycholinguistics.

## 3 Dataset Description

In this work, we study anonymized counseling conversations from a not-for-profit organization providing free crisis intervention via SMS messages. Text-based counseling conversations are particularly well suited for conversation analysis because all interactions between the two dialogue partners are fully observed (*i.e.*, there are no non-textual or non-verbal cues). Moreover, the conversations are important, constrained to dialogue between two people, and outcomes can be clearly defined (*i.e.*, we follow up with the conversation partner as to whether they feel better afterwards), which enables the study of how conversation features are associated with actual outcomes.

### Counseling Process

Any person in distress can text the organization’s public number. Incoming requests are put into a queue and an available counselor picks the request from the queue and engages with the incoming conversation. We refer to the crisis counselor as the *counselor* and the person in distress as the *texter*. After the conversation ends, the texter receives a follow-up question (“How are you feeling now? Better, same, or worse?”) which we use as our conversation quality ground-truth (we use binary labels: good versus same/worse, since we care about improving the situation). In contrast to previous work that has used human judges to rate a caller’s crisis state (Kalafat et al., 2007), we directly obtain this feedback from the texter. Furthermore, the counselor fills out a post-conversation report (*e.g.*, suicide risk, main issue such as depression, relationship, self-harm, suicide, *etc.*). All crisis counselors receive extensive training and commit to weekly shifts for a full year.

### Dataset Statistics

Our dataset contains 408 counselors and 3.2 million messages in 80,885 conversations between November 2013 and November 2014 (see Table 1). All system messages (*e.g.*, instructions), as well as texts that contain survey responses (revealing the ground-truth label for the conversation) were filtered out. Out of these conversations, we use the 15,555, or 19.2%, that contain a ground-truth label (whether the texter feels better or the same/worse after the conversation) for the following analyses. Conversations span a variety of issues of different difficulties (see rows one and two of Table 2). Approval to analyze the dataset was obtained from the Stanford IRB.

## 4 Defining Counseling Quality

The primary goal of this paper is to study strategies that lead to conversations with positive outcomes. Thus, we require a ground-truth notion of conversation quality. In principle, we could study individual conversations and aim to understand what factors make the conversation partner (texter) feel better. However, it is advantageous to focus on the conversation actor (counselor) instead of individual conversations.

There are several benefits of focusing analyses on counselors (rather than individual conversations): First, we are interested in general conversation strategies rather than properties of main issues (*e.g.*, depression vs. suicide). While each conversation is different and will revolve around its main issue, we assume that counselors have a particular style and strategy that is invariant across conversations. Second, we assume that conversation quality is noisy. Even a very good counselor will face some hard conversations in which they do everything right but are still unable to make their conversation partner feel better. Over time, however, the “true” quality of the counselor will become apparent. Third, our goal is to understand successful conversation strategies and to make use of these insights in counselor training. Focusing on the counselor is helpful in understanding, monitoring, and improving counselors’ conversation strategies.

### More vs. Less Successful Counselors

**Figure 1:**
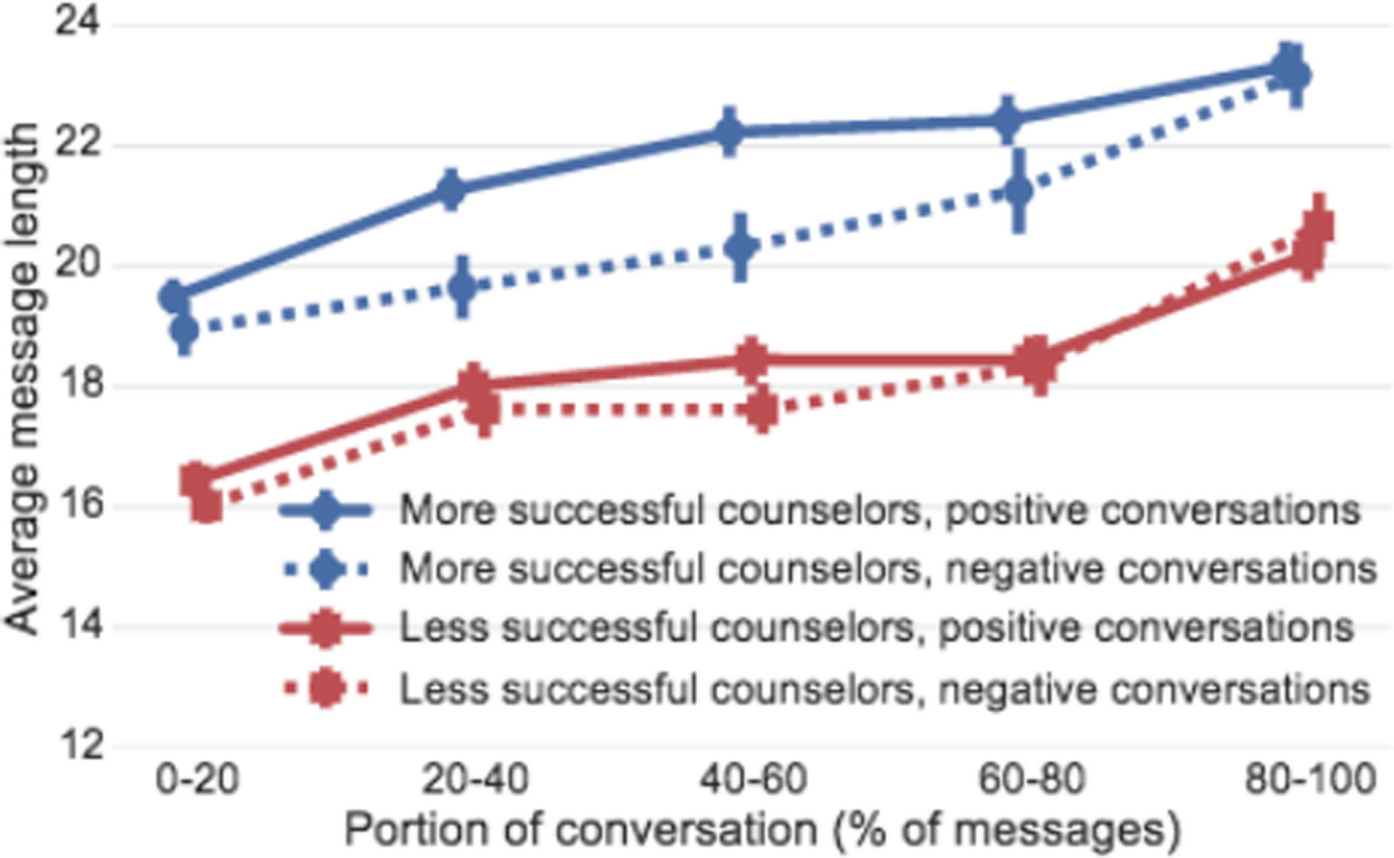
Differences in counselor message length (in #tokens) over the course of the conversation are larger between more and less successful counselors (blue circle/red square) than between positive and negative conversations (solid/dashed). Error bars in all plots correspond to bootstrapped 95% confidence intervals using the member bootstrapping technique from Ren et al. (2010).

We split the counselors into two groups and then compare their behavior. Out of the 113 counselors with more than 15 labeled conversations of at least 30 messages each, we use the most successful 40 counselors as “more successful” counselors and the bottom 40 as “less successful” counselors. Their average success rates are 66.3–85.5% and 42.1–58.6%, respectively. While the counselor-level analysis is of primary concern, we will also differentiate between counselor behavior in “positive” versus “negative” conversations (*i.e.*, those that will eventually make the texter feel better vs. not). Thus, in the remainder of the paper we differentiate between more vs. less successful counselors and positive vs. negative conversations. Studying the cross product of counselors and conversations allows us to gain insights on how both groups behave in positive and negative conversations. For example, Figure 1 illustrates why differentiating between counselors and as well as conversations is necessary: differences in counselor message length over the course of the conversation are bigger between more and less successful counselors than between positive and negative conversations.

### Initial Analysis

Before focusing on detailed analyses of counseling strategies we address two important questions: Do counselors specialize in certain issues? And, do successful counselors appear successful only because they handle “easier” cases?

To gain insights into the “specialization hypothesis” we make use the counselor annotation of the main issue (depression, self-harm, *etc.*). We compare success rates of counselors across different issues and find that successful counselors have a higher fraction of positive conversations across all issues and that less successful counselors typically do not excel at a particular issue. Thus, we conclude that counseling quality is a general trait or skill and supporting that the split into more and less successful counselors is meaningful.

Another simple explanation of the differences between more and less successful counselors could be that successful counselors simply pick “easy” issues. However, we find that this is not the case. In particular, we find that both counselor groups are very similar in how they select conversations from the queue (picking the top-most in 60.1% vs. 60.3%, respectively), work similar shifts, and handle a similar number of conversations simultaneously (1.98 vs. 1.83). Further, we find that both groups face similar distributions of issues over time (see Table 2). We attribute the largest difference, “NA” (main issue not reported), to the more successful counselors being more diligent in filling out the post-conversation report and having fewer conversations that end before the main issue is introduced.

## 5 Counselor Adaptability

In the remainder of the paper we focus on factors that mediate the outcome of a conversation. First, we examine whether successful counselors are more aware that their current conversation is going well or badly and study how the counselor adapts to the situation. We investigate this question by looking for language differences between positive and negative conversations. In particular, we compute a distance measure between the language counselors use in positive conversations and the language counselors use in negative conversations and observe how this distance changes over time.

We capture the time dimension by breaking up each conversation into five even chunks of messages. Then, for each set of counselors (more successful or less successful), conversation outcome (positive or negative), and chunk (first 20%, second 20%, etc.), we build a TF-IDF vector of word occurrences to represent the language of counselors within this subset. We use the global inverse document (*i.e.*, conversation) frequencies instead of the ones from each subset to make the vectors directly comparable and control for different counselors having different numbers of conversations by weighting conversations so all counselors have equal contributions. We then measure the difference between the “positive” and “negative” vector representations by taking the cosine distance in the induced vector space. We also explored using Jensen-Shannon divergence between traditional probabilistic language models and found these methods gave similar results.

### Results

**Figure 2:**
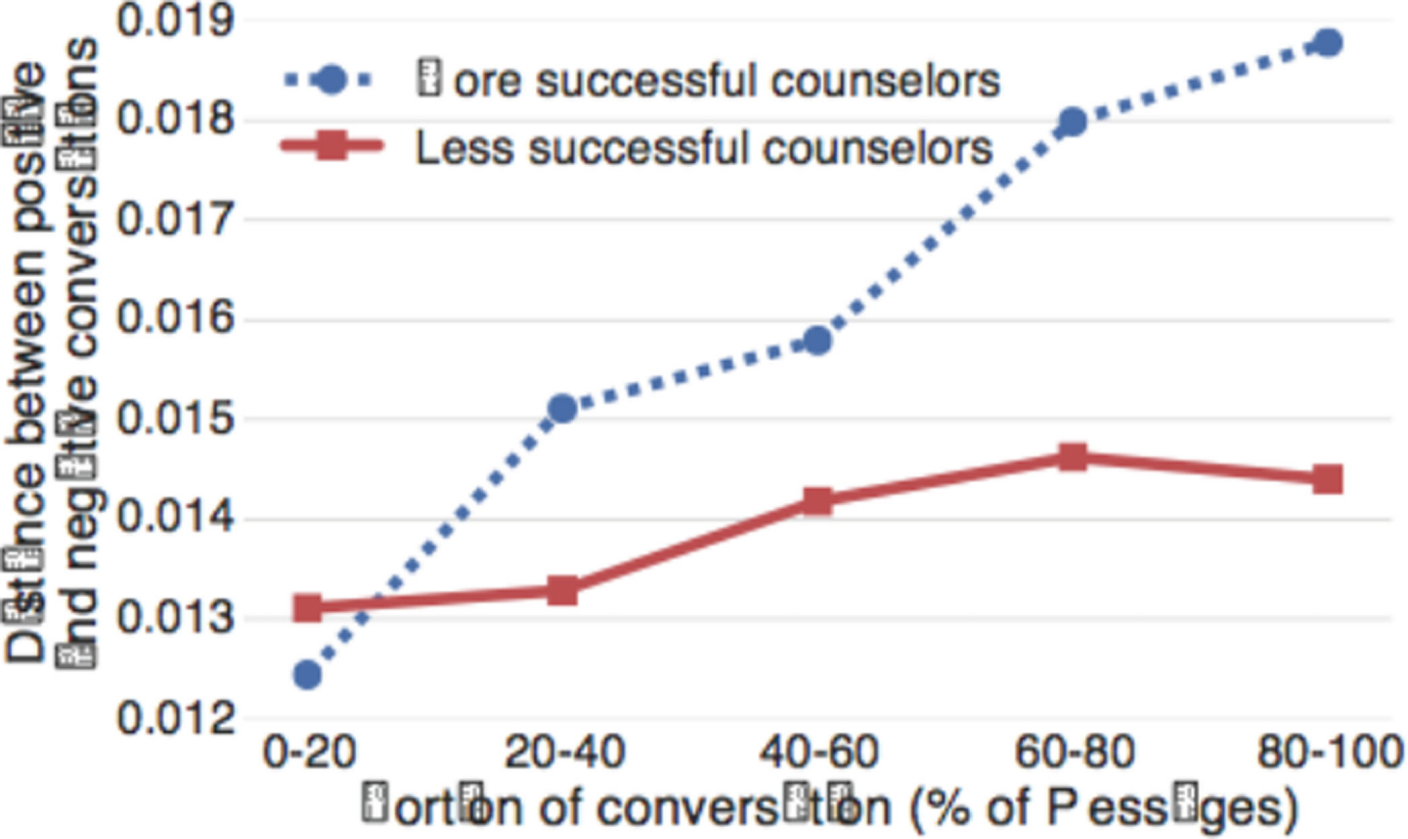
More successful counselors are more varied in their language across positive/negative conversations, suggesting they adapt more. All differences between more successful and less successful counselors except for the 0–20 bucket were found to be statistically significant (p < 0.05; bootstrap resampling test).

We find more successful counselors are more sensitive to whether the conversation is going well or badly and vary their language accordingly (Figure 2). At the beginning of the conversation, the language between positive and negative conversations is quite similar, but then the distance in language increases over time. This increase in distance is much larger for more successful counselors than less successful ones, suggesting they are more aware of when conversations are going poorly and adapt their counseling more in an attempt to remedy the situation.

## 6 Reacting to Ambiguity

Observing that successful counselors are better at adapting to the conversation, we next examine *how* counselors differ and what factors determine the differences. In particular, domain experts have suggested that more successful counselors are better at handling ambiguity in the conversation (Levitt and Jacques, 2005). Here, we use *ambiguity* to refer to the uncertainty of the situation and the texter’s actual core issue resulting from insufficiently short or uncertain descriptions. Does initial ambiguity of the situation negatively affect the conversation? How do more successful counselors deal with ambiguous situations?

### Ambiguity

Throughout this section we measure ambiguity in the conversation as the shortness of the texter’s responses in number of words. While ambiguity could also be measured through concreteness ratings of the words in each message (*e.g.*, using concreteness ratings from Brysbaert et al. (2014)), we find that results are very similar and that length and concreteness are strongly related and hard to distinguish.

### 6.1 Initial Ambiguity and Situation Setter

It is challenging to measure ambiguity and reactions to ambiguity at arbitrary points throughout the conversation since it strongly depends on the context of the entire conversation (*i.e.*, all earlier messages and questions). However, we can study nearly identical *beginnings* of conversations where we can directly compare how more successful and less successful counselors react given nearly identical situations (the texter first sharing their reason for texting in). We identify the *situation setter* within each conversation as the first long message by the texter (typically a response to a “Can you tell me more about what is going on?” question by the counselor).

#### Results

**Figure 3:**
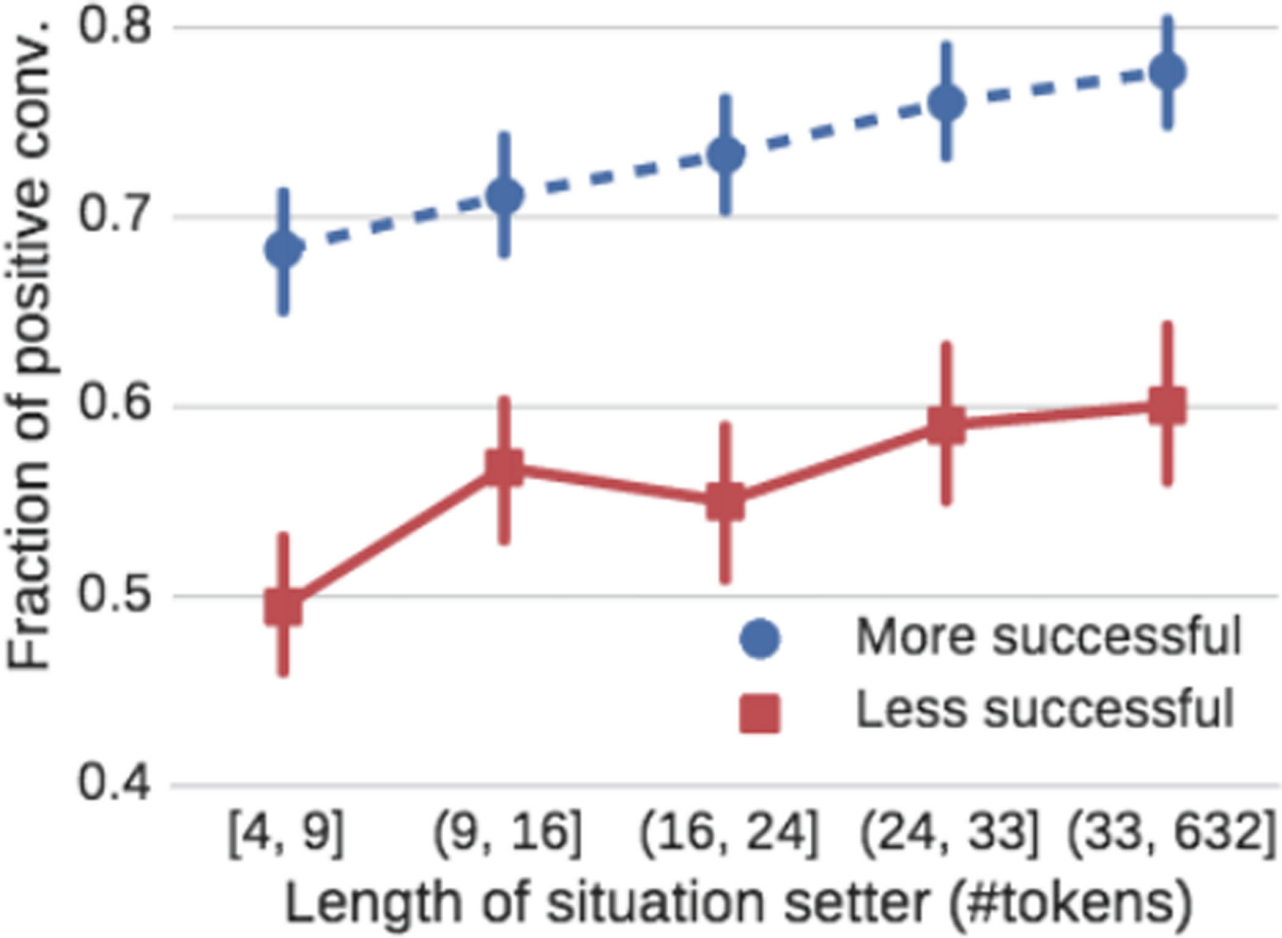
More ambiguous situations (length of situation setter) are less likely to result in positive conversations.

**Figure 4:**
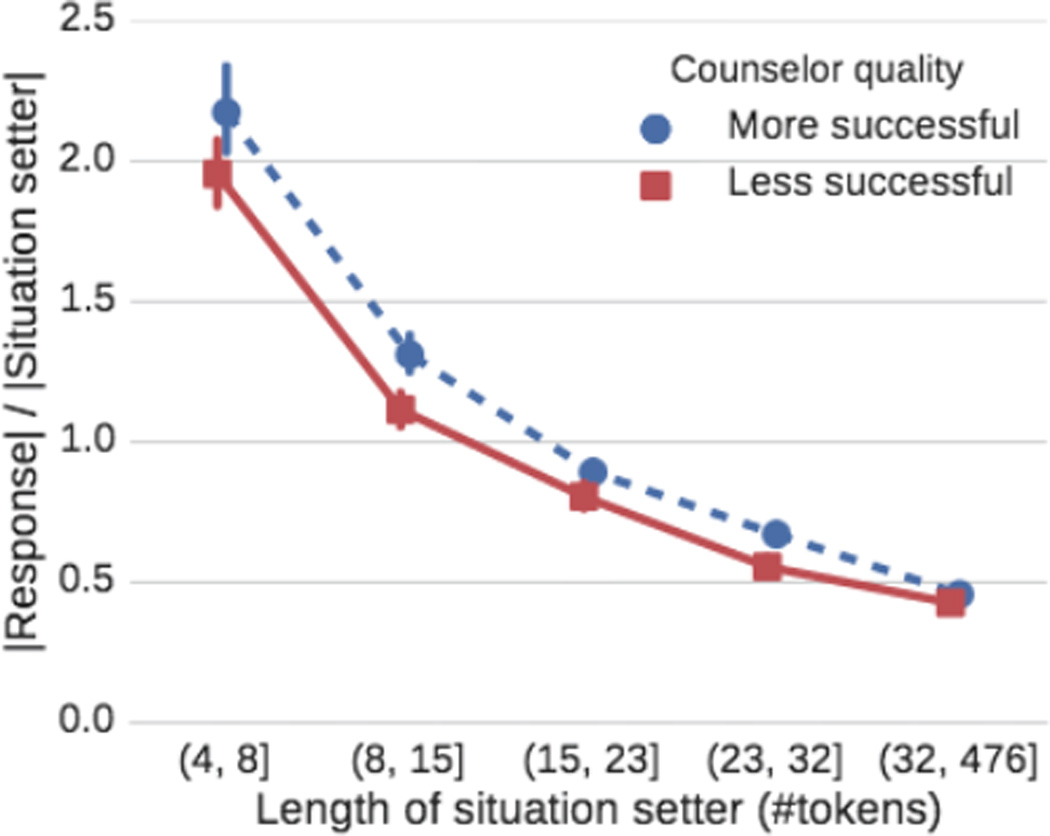
All counselors react to short, ambiguous messages by writing more (relative to the texter message) but more successful counselors do it more than less successful counselors.

We find that ambiguity plays an important role in counseling conversations. Figure 3 shows that more ambiguous situations (shorter length of situation setter) are less likely to result in successful conversations (we obtain similar results when measuring concreteness (Brysbaert et al., 2014) directly). Further, we find that counselors generally react to short and ambiguous situation setters by writing significantly more than the texters (Figure 4; if counselors wrote exactly as much as the texter, we would expect a horizontal line *y* = 1). However, more successful counselors react more strongly to ambiguous situations than less successful counselors.

### 6.2 How to Respond to Ambiguity

Having observed that ambiguity plays an important role in counseling conversations, we now examine in greater detail how counselors respond to nearly identical situations.

We match situation setters by representing them through TF-IDF vectors on bigrams and find similar situation setters as nearest neighbors within a certain cosine distance in the induced space.^1^ We only consider situation setters that are part of a dense cluster with at least 10 neighbors, allowing us to compare follow-up responses by the counselors (4829/12770 situation setters were part of one of 589 such clusters). We also used distributed word embeddings (*e.g.*, (Mikolov et al., 2013)) instead of TF-IDF vectors but found the latter to produce better clusters.

Based on counselor training materials we hypothesize that more successful counselors

- address ambiguity by writing more themselves,
- use more check questions (statements that tell the conversation partner that you understand them while avoiding the introduction of any opinion or advice (Labov and Fanshel, 1977); *e.g*.”that sounds like…”),
- check for suicidal thoughts early (*e.g.*, “want to die”),
- thank the texter for showing the courage to talk to them (*e.g.*, “appreciate”),
- use more hedges (mitigating words used to lessen the impact of an utterance; *e.g.*, “maybe”, “fairly”),
- and that they are less likely to respond with surprise (*e.g.*, “oh, this sounds really awful”).

A set of regular expressions is used to detect each class of responses (similar to the examples above).

#### Results

We find several statistically significant differences in how counselors respond to nearly identical situation setters (see Table 3). While situation setters tend to be slightly longer for more successful counselors (suggesting that conversations are not perfectly randomly assigned), counselor responses are significantly longer and also spur longer texter responses. Further, the more successful counselors respond in a way that is less similar to the original situation setter (measured by cosine similarity in TF-IDF space) compared to less successful counselors (but the texter’s response does not seem affected). We do find that more successful counselors use more check questions, check for suicide ideation more often, show the texter more appreciation, and use more hedges, but we did not find a significant difference with respect to responding with surprise.

### 6.3 Response Templates and Creativity

In Section 6.2, we observed that more successful counselors make use of certain templates (including check questions, checks for suicidal thoughts, affirmation, and using hedges). While this could suggest that counselors should stick to such predefined templates, we find that, in fact, more successful counselors do respond in more creative ways.

We define a measure of how “templated” the counselors responses are by counting the number of similar responses in TF-IDF space for the counselor reaction (*c.f.*, Section 6.2; again using a manually defined and validated threshold on cosine distance).

**Figure 5:**
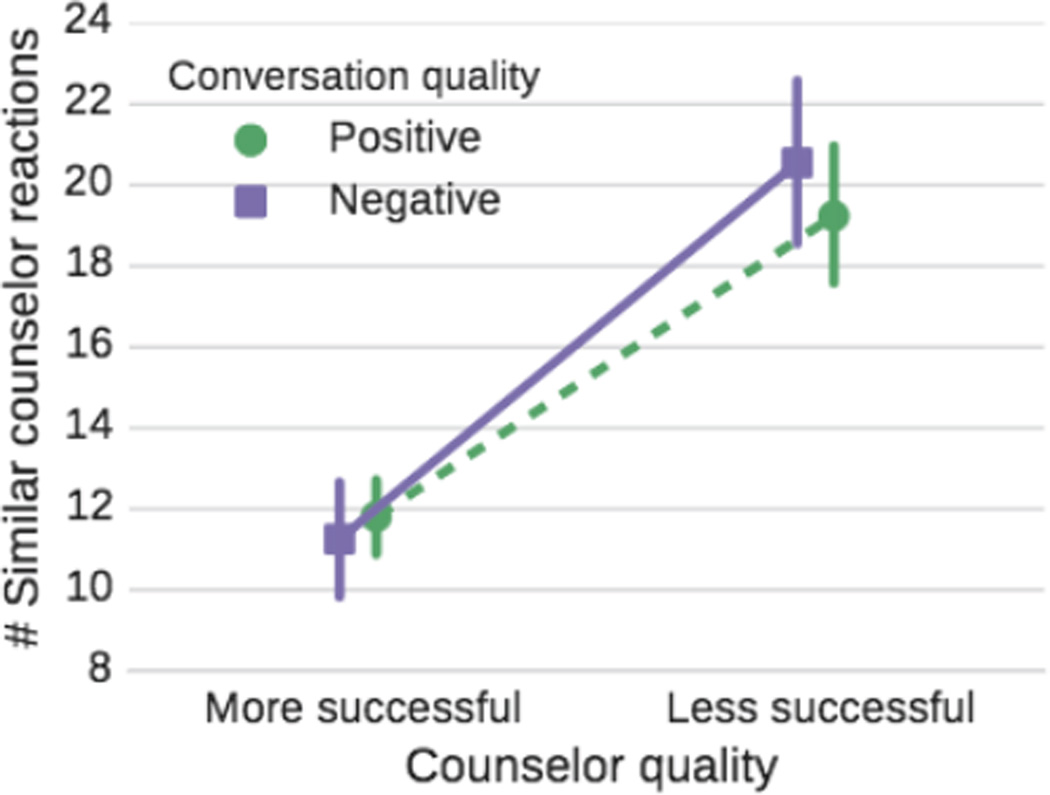
More successful counselors use less common/templated responses (after the texter first explains the situation). This suggests that they respond in a more creative way. There is no significant difference between positive and negative conversations.

Figure 5 shows that more successful counselors use less common/templated questions. This suggests that while more successful counselors questions follow certain patterns, they are more *creative* in their response to each situation. This tailoring of responses requires more effort from the counselor, which is consistent with the results in Figure 1 that showed that more successful counselors put in more effort in composing longer messages as well.

## 7 Ensuring Conversation Progress

After demonstrating content-level differences between counselors, we now explore temporal differences in how counselors progress through conversations. Using an unsupervised conversation model, we are able to discover distinct conversation stages and find differences between counselors in how they move through these stages. We further provide evidence that these differences could be related to power and authority by measuring linguistic coordination between the counselor and texter.

### 7.1 Unsupervised Conversation Model

Counseling conversations follow a common structure due to the nature of conversation as well as counselor training. Typically, counselors first introduce themselves, get to know the texter and their situation, and then engage in constructive problem solving. We employ unsupervised conversation modeling techniques to capture this stage-like structure within conversations.

**Figure 6:**
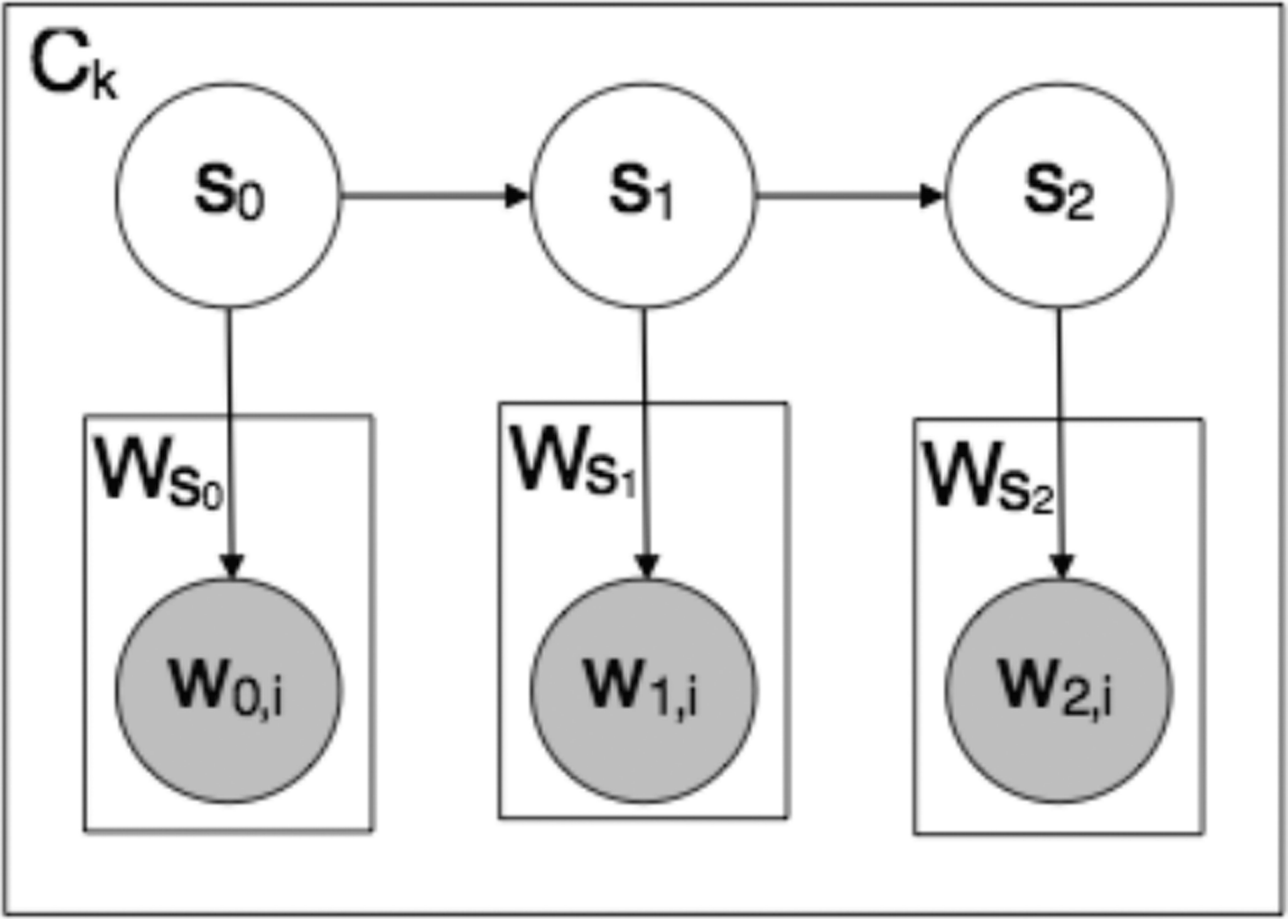
Our conversation model generates a particular conversation C_k_ by first generating a sequence of hidden states S_0_, S_1_,… according to a Markov model. Each state S_i_ then generates a message as a bag of words W_i, 0_, W_i, 1_ … according a unigram language model W_S_i__.

**Figure 7:**
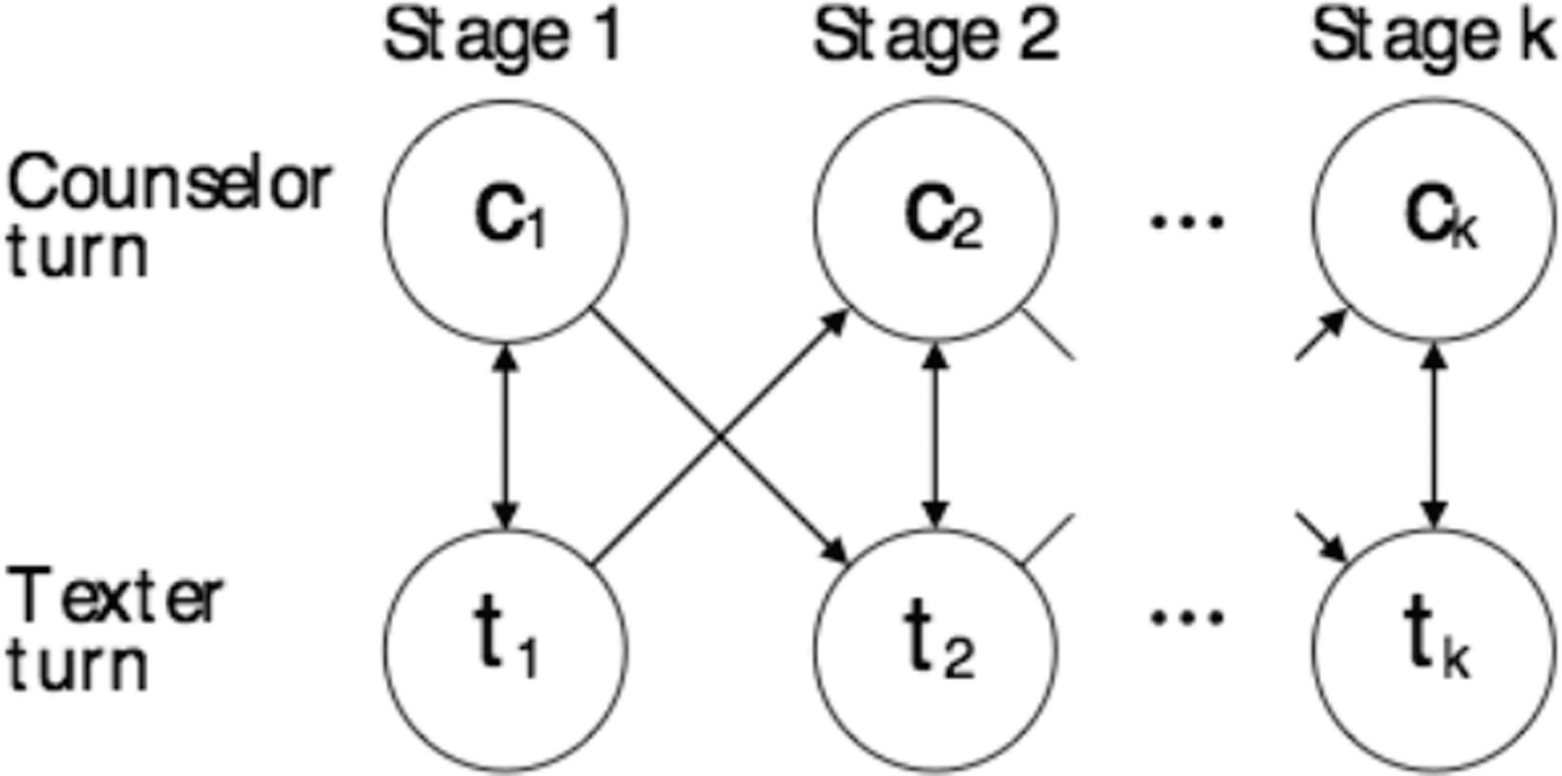
Allowed state transitions for the conversation model. Counselor and texter messages are produced by distinct states and conversations must progress through the stages in increasing order.

Our conversation model is a message-level Hidden Markov Model (HMM). Figure 6 illustrates the basic model where hidden states of the HMM represent *conversation stages*. Unlike in prior work on conversation modeling, we impose a fixed ordering on the stages and only allow transitions from the current stage to the next one (Figure 7). This causes it to learn a fixed dialogue structure common to all of the counseling sessions as opposed to conversation topics. Furthermore, we separately model counselor and texter messages by treating their turns in the conversation as distinct states. We train the conversation model with expectation maximization, using the forward-backward algorithm to produce the distributions during each expectation step. We initialized the model with each stage producing messages according to a unigram distribution estimated from all messages in the dataset and uniform transition probabilities. The unigram language models are defined over all words occurring more than 20 times (over 98% of words in the dataset), with other words replaced by an unknown token.

#### Results

We explored training the model with various numbers of stages and found five stages to produce a distinct and easily interpretable representation of a conversation’s progress. Table 4 shows the words most unique to each stage. The first and last stages consist of the basic introductions and wrap-ups common to all conversations. In stage 2, the texter introduces the main issue, while the counselor asks for clarifications and expresses empathy for the situation. In stage 3, the counselor and texter discuss the problem, particularly in relation to the other people involved. In stage 4, the counselor and texter discuss actionable strategies that could help the texter. This is a well-known part of crisis counselor training called “collaborative problem solving.”

### 7.2 Analyzing Counselor Progression

Do counselors differ in how much time they spend at each stage? In order to explore how counselors progress through the stages, we use the Viterbi algorithm to assign each conversation the most likely sequence of stages according to our conversation model. We then compute the average duration in messages of each stage for both more and less successful counselors. We control for the different distributions of positive and negative conversations among more successful and less successful counselors by giving the two classes of conversations equal weight and control for different conversation lengths by only including conversations between 40 and 60 messages long.

#### Results

**Figure 8:**
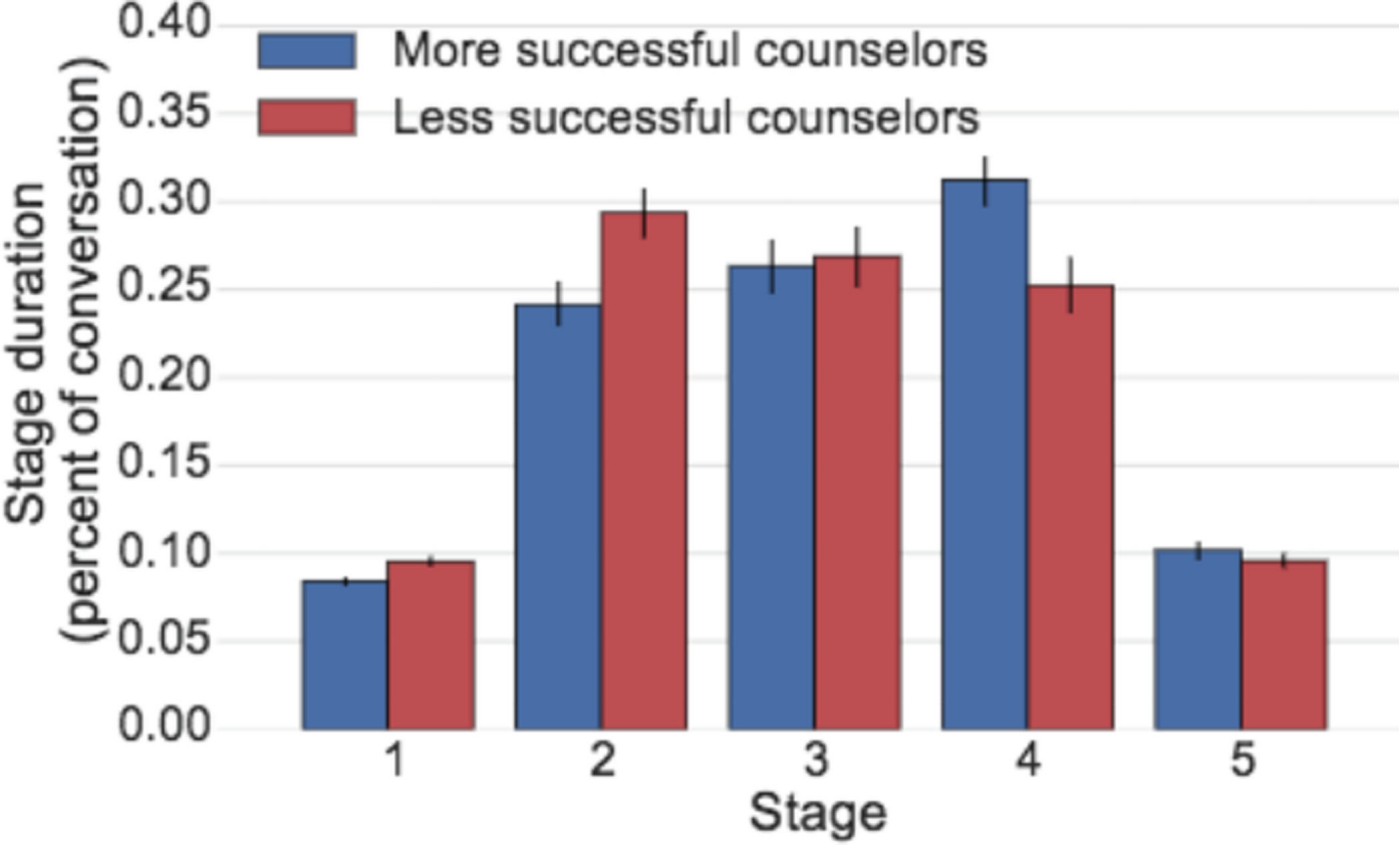
More successful counselors are quicker to get to know texter and issue (stage 2) and use more of their time in the “problem solving” phase (stage 4).

We find that more successful counselors are quicker to move past the earlier stages, particularly stage 2, and spend more time in later stages, particularly stage 4 (Figure 8). This suggests they are able to more quickly get to know the texter and then spend more time in the problem solving phase of the conversation, which could be one of the reasons they are more successful.

### 7.3 Coordination and Power Differences

One possible explanation for the more successful counselors’ ability to quickly move through the early stages is that they have more “power” in the conversation and can thus exert more control over the progression of the conversation. We explore this idea by analyzing linguistic coordination, which measures how much the conversation partners adapt to each other’s conversational styles. Research has shown that conversation participants who have a greater position of power coordinate less (*i.e.*, they do not adapt their linguistic style to mimic the other conversational participant as strongly) (Danescu-Niculescu-Mizil et al., 2012).

In our analysis, we use the “Aggregated 2” coordination measure *C*(*B*, *A*) from Danescu-Niculescu-Mizil (2012), which measures how much group *B* coordinates to group *A* (a higher number means more coordination). The measure is computed by counting how often specific markers (e.g., auxiliary verbs) are exhibited in conversations. If someone tends to use a particular marker right after their conversation partner uses that marker, it suggests they are coordinating to their partner.

Formally, let set *S* be a set of exchanges, each involving an initial utterance *u*_1_ by *a* ∈ *A* and a reply *u*_2_ by *b* ∈ *B*. Then the coordination of *b* to *A* according to a linguistic marker *m* is:

$$C^{m}(b,A)=P(\varepsilon_{u_{2}\to u_{1}}^{m}|\varepsilon_{u_{1}}^{m})-P(\varepsilon_{u_{2}\to u_{1}}^{m})$$

where $\varepsilon_{u_{1}}^{m}$ is the event that utterance *u*_1_ exhibits *m* (*i.e.*, contains a word from category *m*) and $\varepsilon_{u_{2}\to u_{1}}^{m}$ is the event that reply *u*_2_ to *u*_1_ exhibits *m*. The probabilities are estimated across all exchanges in *S*. To aggregate across different markers, we average the coordination values of *C^m^*(*b*, *A*) over all markers *m* to get a macro-average *C*(*b*, *A*). The coordination between groups *B* and *A* is then defined as the mean of the coordinations of all members of group *B* towards the group *A*.

We use eight markers from Danescu-Niculescu-Mizil (2012), which are considered to be processed by humans in a generally non-conscious fashion: articles, auxiliary verbs, conjunctions, high-frequency adverbs, indefinite pronouns, personal pronouns, prepositions, and quantifiers.

#### Results

Texters coordinate less than counselors, with texters having a coordination value of *C*(texter, counselor)=0.019 compared to the counselor’s *C*(counselor, texter) =0.030, suggesting that the texters hold more “power” in the conversation. However, more successful counselors coordinate less than less successful ones (*C*(more succ. counselors, texter)=0.029 vs. *C*(less succ. counselors, texter)=0.032). All differences are statistically significant (*p* < 0.01; Mann-Whitney U test). This suggests that more successful counselors act with more control over the conversation, which could explain why they are quicker to make it through the initial conversation stages.

## 8 Facilitating Perspective Change

Thus far, we have studied conversation dynamics and their relation to conversation success from the counselor perspective. In this section, we show that *perspective change* in the *texter* over time is associated with a higher likelihood of conversation success. Prior work has shown that day-to-day changes in writing style are associated with positive health outcomes (Campbell and Pennebaker, 2003), and existing theories link depression to a negative view of the future (Pyszczynski et al., 1987) and a self-focusing style (Pyszczynski and Greenberg, 1987). Here, we propose a novel measure to quantify three orthogonal aspects of perspective change within a single conversation: *time, self*, and *sentiment*. Further, we show that the counselor might be able to actively induce perspective change.

### Time

**Figure 9:**
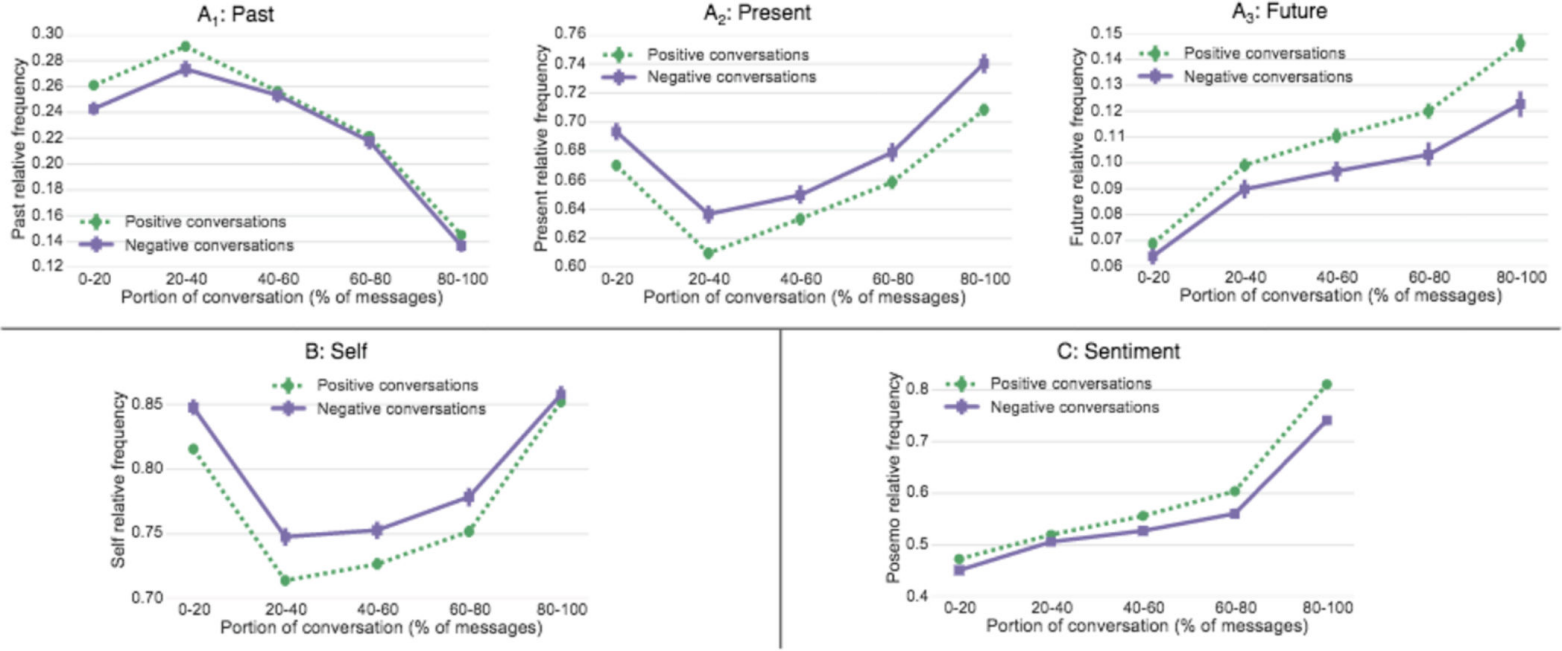
A: Throughout the conversation there is a shift from talking about the past to future, where in positive conversations this shift is greater; B: Texters that talk more about others more often feel better after the conversation; C: More positive sentiment by the texter throughout the conversation is associated with successful conversations.

Texters start explaining their issue largely in terms of the past and present but over time talk more about the future (see Figure 9A; each plot shows the relative amount of words in the LIWC past, present, and future categories (Tausczik and Pennebaker, 2010)). We find that texters writing more about the future are more likely to feel better after the conversation. This suggests that changing the perspective from issues in the past towards the future is associated with a higher likelihood of successfully working through the crisis.

### Self

Another important aspect of behavior change is to what degree the texter is able to change their perspective from talking about themselves to considering others and potentially the effect of their situation on others (Pyszczynski and Greenberg, 1987; Campbell and Pennebaker, 2003). We measure how much the texter is focused on themselves by the relative amount of first person singular pronouns (I, me, mine) versus third person singular/plural pronouns (she, her, him / they, their), again using LIWC. Figure 9B shows that a smaller amount of self-focus is associated with more successful conversations (providing support for the self-focus model of depression (Pyszczynski and Greenberg, 1987)). We hypothesize that the lack of difference at the end of the conversation is due to conversation norms such as thanking the counselor (‘*I* really appreciate it.”) even if the texter does not actually feel better.

### Sentiment

Lastly, we investigate how much a change in sentiment of the texter throughout the conversation is associated with conversation success. We measure sentiment as the relative fraction of positive words using the LIWC PosEmo and NegEmo sentiment lexicons. The results in Figure 9C show that texters always start out more negative (value below 0.5), but that the sentiment becomes more positive over time for both positive and negative conversations. However, we find that the separation between both groups grows larger over time, which suggests that a positive perspective change throughout the conversation is related to higher likelihood of conversation success. We find that both curves increase significantly at the very end of the conversation. Again, we attribute this to conversation norms such as thanking the counselor for listening even when the texter does not actually feel better. Together with the result on talking about the future, these findings are consistent with the theory of Pyszczynski et al. (1987) that depression is related to a negative view of the future.

### Role of a Counselor

Given that positive conversations often exhibit perspective change, a natural question is how counselors can encourage perspective change in the texter. We investigate this by exploring the hypothesis that the texter will tend to talk more about something (*e.g.*, the future), if the counselor first talks about it. We measure this tendency using the same coordination measures as Section 7.3 except that instead of using stylistic LIWC markers (*e.g.*, auxiliary verbs, quantifiers), we use the LIWC markers relevant to the particular aspect of perspective change (e.g., Future, HeShe, PosEmo). In all cases we find a statistically significant (p < 0.01; Mann-Whitney U-test) increase in the likelihood of the texter using a LIWC marker if the counselor used it in the previous message (~4–5% change). This link between perspective change and how the counselor conducts the conversation suggests that the counselor might be able to actively induce measurable perspective change in the texter.

## 9 Predicting Counseling Success

In this section, we combine our quantitative insights into a prediction task. We show that the linguistic aspects of crisis counseling explored in previous sections have predictive power at the level of individual conversations by evaluating their effectiveness as features in classifying the outcome of conversations. Specifically, we create a balanced dataset of positive and negative conversations more than 30 messages long and train a logistic regression model to predict the outcome given the first *x*% of messages in the conversation. There are 3619 such negative conversations and and we randomly subsample the larger set of positive conversations. We train the model with batch gradient descent and use L1 regularization when n-gram features are present and L2 regularization otherwise. We evaluate our model with 10-fold cross-validation and compare models using the area under the ROC curve (AUC).

### Features

We include three aspects of counselor messages discussed in Section 6: hedges, check questions, and the similarity between the counselor’s message and previous texter message. We add a measure of how much progress the counselor has made (Section 7) by computing the Viterbi path of stages for the conversation (only for the first *x*%) with the HMM conversation model and then adding the duration of each stage (in #messages) as a feature. Additionally, we add average message length and average sentiment per message using VADER sentiment (Hutto and Gilbert, 2014). Further, we add temporal dynamics to the model by adding feature conjunctions with the stages HMM model. After running the stages model over the *x*% of the conversation available to the classifier, we add each feature’s average value over each stage as additional features. Lastly, we explore the benefits of adding surface-level text features to the model by adding unigram and bigram features. Because the focus of this work is on counseling strategies, we primarily experiment with models using only features from counselor messages. For completeness, we also report results for a model including texter features.

### Prediction Results

**Figure 10:**
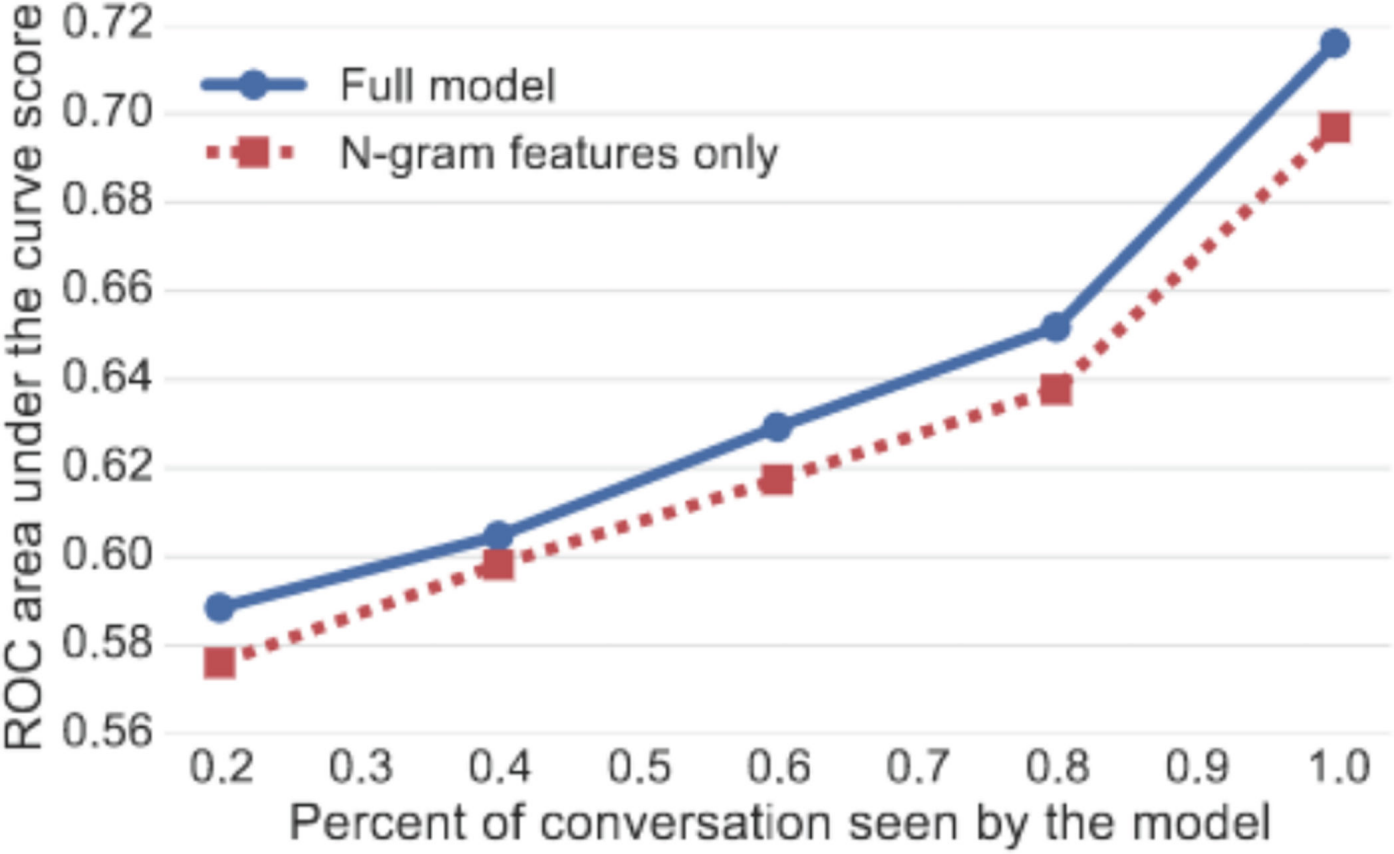
Prediction accuracies vs. percent of the conversation seen by the model (without texter features).

The model’s accuracy increases with *x*, and we show that the model is able to distinguish positive and negative conversations after only seeing the first 20% of the conversation (see Figure 10). We attribute the significant increase in performance for x = 100 (Accuracy=0.687, AUC=0.716) to strong linguistic cues that appear as a conversation wraps up (e.g., “I’m glad you feel better.”). To avoid this issue, our detailed feature analysis is performed at x = 80.

### Feature Analysis

The model performance as features are incrementally added to the model is shown in Table 5. All features improve model accuracy significantly (p < 0.001; paired bootstrap resampling test). Adding n-gram features produces the largest boost in AUC and significantly improves over a model just using n-gram features (0.638 vs. 0.652 AUC). Note that most features in the full model are based on word frequency counts that can be derived from n-grams which explains why a simple n-gram model already performs quite well. However, our model performs well with only a small set of linguistic features, demonstrating they provide a substantial amount of the predictive power. The effectiveness of these features shows that, in addition to exhibiting group-level differences reported earlier in this paper, they provide useful signal for predicting the outcome of individual conversations.

## 10 Conclusion & Future Work

Knowledge about how to conduct a successful counseling conversation has been limited by the fact that studies have remained largely qualitative and small-scale. In this work, we presented a large-scale quantitative study on the discourse of counseling conversations. We developed a set of novel computational discourse analysis methods suited for large-scale datasets and used them to discover actionable conversation strategies that are associated with better conversation outcomes. We hope that this work will inspire future generations of tools available to people in crisis as well as their counselors. For example, our insights could help improve counselor training and give rise to real-time counseling quality monitoring and answer suggestion support tools.

## Figures and Tables

**Table 1:** Basic dataset statistics.

Dataset statistics	
Conversations	80,885
Conversations with survey response	15,555 (19.2%)
Messages	3.2 million
Messages with survey response	663,026 (20.6%)
Counselors	408
Messages per conversation*	42.6
Words per message*	19.2

Rows marked with * are computed over conversations with survey responses.

**Table 2:** Frequencies and success rates for the nine most common conversation issues (NA: Not available). On average, more and less successful counselors face the same distribution of issues.

	NA	Depressed	Relationship	Self harm	Family	Suicide	Stress	Anxiety	Other
Success rate	0.556	0.612	0.659	0.672	0.711	0.573	0.696	0.671	0.537
Frequency	0.200	0.200	0.089	0.074	0.071	0.063	0.041	0.039	0.035
Frequency with more successful counselors	0.203	0.199	0.089	0.067	0.072	0.061	0.048	0.042	0.030
Frequency with less successful counselors	0.223	0.208	0.087	0.070	0.067	0.056	0.030	0.032	0.028

**Table 3:** Differences between more and less successful counselors (C; More S. and Less S.) in responses to nearly identical situation setters (Sec. 6.1) by the texter (T).

	More S.	Less S.	Test
% conversations successful	70.7	51.7	***
#messages in conversation	57.0	46.7	***
Situation setter length (#tokens)	12.1	10.7	***
C response length (#tokens)	15.8	11.8	***
T response length (#tokens)	20.4	18.8	***
% Cosine sim. C resp. to context	11.9	14.8	***
% Cosine sim. T resp. to context	7.6	7.3	–
% C resp. w check question	12.6	4.1	***
% C resp. w suicide check	13.5	10.3	***
% C resp. w thanks	6.3	2.4	***
% C resp. w hedges	41.4	36.8	***
% C resp. w surprise	3.3	2.8	–

Last column contains significance levels of Wilcoxon Signed Rank Tests (*** *p* < 0.001, – *p* > 0.05).

**Table 4:** The top 5 words for counselors and texters with greatest increase in likelihood of appearing in each stage. The model successfully identifies interpretable stages consistent with counseling guidelines (qualitative interpretation based on stage assignment and model parameters; only words occurring more than five hundred times are shown).

Stage	Interpretation	Top words for texter	Top words for counselor
1	Introductions	hi, hello, name, listen, hey	hi, name, hello, hey, brings
2	Problem introduction	dating, moved, date, liked, ended	gosh, terrible, hurtful, painful, ago
3	Problem exploration	knows, worry, burden, teacher, group	react, cares, considered, supportive, wants
4	Problem solving	write, writing, music, reading, play	hobbies, writing, activities, distract, music
5	Wrap up	goodnight, bye, thank, thanks, appreciate	goodnight, 247, anytime, luck, 24

**Table 5:** Performance of nested models predicting conversation outcome given the first 80% of the conversation. In bold: full models with only counselor features and with additional texter features.

Features	ROC AUC
Counselor unigrams only	0.630
Counselor unigrams and bigrams only	0.638
None	0.5
+ hedges	0.514 (+0.014)
+ check questions	0.546 (+0.032)
+ similarity to last message	0.553 (+0.007)
+ duration of each stage	0.561 (+0.008)
+ sentiment	0.590 (+0.029)
+ message length	0.596 (+0.006)
+ stages feature conjunction	0.606 (+0.010)
+ counselor unigrams and bigrams	**0.652 (+0.046)**
+ texter unigrams and bigrams	**0.708 (+0.056)**

## Footnotes

1 Threshold manually set after qualitative analysis of matches from randomly chosen clusters. Results were not overly sensitive to threshold choice, choice of representation (*e.g.*, word vectors), and distance measure (*e.g.*, Euclidean).
